# Personality Traits and Attitudes Toward Traffic Safety Predict Risky Behavior Across Young, Adult, and Older Drivers

**DOI:** 10.3389/fpsyg.2019.00536

**Published:** 2019-03-11

**Authors:** Fabio Lucidi, Laura Girelli, Andrea Chirico, Fabio Alivernini, Mauro Cozzolino, Cristiano Violani, Luca Mallia

**Affiliations:** ^1^Department of Social and Developmental Psychology, La Sapienza University of Rome, Rome, Italy; ^2^Department of Human, Philosophical, and Educational Sciences, University of Salerno, Fisciano, Italy; ^3^National Institute for the Evaluation of the Education System, Rome, Italy; ^4^Department of Psychology, La Sapienza University of Rome, Rome, Italy; ^5^Department of Movement, Human and Health Sciences, Foro Italico University of Rome, Rome, Italy

**Keywords:** attitudes, personality, age, driving violations, driving errors, driving lapses

## Abstract

In the last few decades, several studies have investigated the role of personality traits and attitudes toward traffic safety in predicting driving behaviors in diverse types of drivers across several countries. However, to the best of our knowledge, no studies so far have investigated the possible moderating role played by age in relation to predictors of accident risk. Answering this open question would provide information about the generalizability of the model across different subpopulations and would make possible the tailoring of the interventions to specific target groups. The study involved 1,286 drivers from three different age groups (young: *n* = 435; adult: *n* = 412; old: *n* = 439) which completed a questionnaire measuring drivers’ personality traits (i.e., anxiety, hostility, excitement seeking, altruism, normlessness), positive attitudes toward traffic safety, risky driving behaviors (i.e., errors, lapses, and traffic violations), accident involvement and number of traffic fines issued in the last 12 months. Multi-group Variance Based Structural Equation Modeling (VB-SEM) across the three age groups showed that the hypothesized model had a good fit with the data in all the three age groups. However, some pattern of relationships between the variables varied across the three groups, for example, if considering the direct effects of personality traits on risky driving behaviors, anxiety, altruism, and normlessness predicted violations only in young and adult drivers, whereas excitement seeking was associated with lapses only in young drivers; anxiety was a positive predictor of drivers’ errors, both in adult and older drivers, whereas excitement seeking predicted errors in adult and young drivers. On the other hand, attitudes significantly and negatively predicted violations and errors in all the three age groups, whereas they significantly and negatively predicted lapses only in young and older drivers. The results of the present study provided empirical basis to develop evidence-based road safety interventions differently tailored to the specific life’s stage of the drivers.

## Introduction

According to the World Health Organization [WHO] (2018), every year around the world 1.25 million people die because of a road traffic accident and, between 20 and 50 million more people are injured with many of them incurring a disability (World Health Organization [WHO], 2018). Epidemiological data showed that young and adult drivers (aged 15–44 years) account for 48% of road traffic deaths worldwide (World Health Organization [WHO], 2018). Additionally, as they become more fragile with aging, drivers aged 65 or older are even more likely to be fatally injured in traffic accidents as compared to younger drivers aged 45–64 years (78 fatalities per million population versus 58 fatalities per million population, Broughton et al., 2012). Thus, today car related accidents represent a huge issue for public health since they involve people from different age groups and represent a high social cost for communities (Wijnen and Stipdonk, 2016).

In the last decades, in order to reduce the risk of car accidents, the psychological literature has paid particular attention to drivers’ behavior at the wheel, since it represents a key factor to look at in order to reduce car accidents (World Health Organization [WHO], 2018). Driving is a complex activity that involves several processes both cognitive and decisional. Thus, the personal factors that influence these processes become crucial to understand. To this end, Reason et al. (1990) taxonomy of driving behaviors represents the widest theoretical model in order to understand the behaviors at the wheel that may be at risk. In their model they categorize three different risky driving behaviors, with each of them related to a distinct cognitive and decisional process: (1) errors, which consists in a failure of planned actions to achieve their intended consequences and largely representing information-processing deficits (e.g., braking too quickly on a slippery road); (2) lapses, which consists in failures of attention and memory (e.g., trying to drive away from traffic lights in third gear); (3) violations, which consists in conscious and deliberate decisions to deviate from rules or safe driving practices (e.g., deciding to do not stop at the red light). The processes underlining these behaviors may develop along the drivers’ life stages (Anstey et al., 2005; Zicat et al., 2018) as well as the extent to which these driving behaviors impact safety on the road (de Winter et al., 2015). Several studies, in fact, have demonstrated that the risky behaviors listed above were differently associated with accident risk in drivers depending on the age (Af Wåhlberg et al., 2015; de Winter et al., 2015). For instance, according to a recent meta-analysis by de Winter and Dodou (2010) the more older were the drivers, the less their violations were predictive of accidents, the more younger were the drivers, the more their violations predicted accidents. Furthermore, errors or lapses did not predict accident involvement for young and adult drivers (Parker et al., 1995a,b), whereas they did in older drivers (e.g., Parker et al., 2000).

In order to understand the personal factors that may explain these differences in crashes risk, several studies have focused on the role of drivers’ personality characteristics in influencing the behavior at the wheel of diverse ages’ drivers (e.g., Iversen and Rundmo, 2002; Yang et al., 2013; Shen et al., 2018), and on the role of other variables, such as attitudes toward traffic safety (Iversen and Rundmo, 2004), that may mediate this relationship. In fact, personality is a quite permanent factor that is not manageable though interventions and that is hypothesized to be a distal predictor of behavior, therefore it can only affect attitudes, which are considered to be the more immediate antecedents of behavioral intention and action (Fishbein and Cappella, 2006). To sustain the crucial role of drivers’ attitudes, Nordfjærn et al. (2010) found that some personality characteristics, such as anxiety, sensation-seeking and normlessness were weakly associated with aberrant driving behavior and demonstrated that the relationship between personality and risky driving could be better understood when considering attitudes toward risky driving.

The association between personality, attitudes, and risky driving behavior has been largely investigated by Ulleberg and Rundmo (2003). In their model, they hypothesized that some personality characteristics of the drivers affected risky driving behaviors, both directly and indirectly, through the effects of attitudes toward traffic safety. In their pioneering study on young drivers, Ulleberg and Rundmo demonstrated that anxiety, hostility, normlessness, excitement-seeking, and aggression were indirectly associated to risky driving through attitudes toward driving safety, whereas altruism was directly associated with risky driving. More recently, other scholars tested this model in different groups of drivers, including also the Reason et al.’s (1990) taxonomy of risky driving (i.e., violations, errors and lapses) as well as indicators of accidents risk and driving rule violations. More specifically, Lucidi et al. (2014) evaluated the validity of Ulleberg and Rundmo’s (2003) model to predict some of the risky driving behaviors (violations, lapses and errors) and to prevent car accidents’ risk in a large sample of older drivers, also taking into consideration the effects of attitudes toward traffic safety. They found that anxiety positively predicted, whereas hostility and normlessness negatively predicted attitudes toward traffic safety, which in turn, affected the different types of risky behaviors at the wheel. Additionally, the study confirmed a direct and positive effect of excitement-seeking upon violations, and a direct and positive association of hostility with both lapses and errors. Lastly, the findings also displayed a strong correlation between the self-reported aberrant driving behaviors and more objective measures of risky driving, for example a higher number of traffic violations was associated with a greater amount of traffic fines received, a larger number of errors was related to a higher likelihood to be involved in a car crash in the previous year. In order to extend and generalize the validity of the model to different types of drivers, Mallia et al. (2015) focused on professional bus drivers. The results showed that altruism, excitement seeking, and normlessness were significantly associated with bus drivers’ attitudes toward traffic safety. As for the study on older drivers (i.e., Lucidi et al., 2014), they found that bus drivers’ positive attitudes toward traffic safety prevented driving violations, lapses and errors, this emphasizing the valuable effects of positive attitudes also in professional drivers. The study also showed that hostility had a positive and direct association both with violations and errors, whereas anxiety was directly and positively related to lapses. Finally, with respect to the validity of the model in predicting risky driving, the study showed that only driving violations predicted accident-risk in older drivers.

Overall, past research confirmed the validity of the Ulleberg and Rundmo’s (2003) model in explaining risky behavior at the wheel in young (Ulleberg and Rundmo, 2003), older (Lucidi et al., 2014), and professional drivers (Mallia et al., 2015). Furthermore, more recent studies (i.e., Lucidi et al., 2014; Mallia et al., 2015) addressed how this model could be integrated with self-reported behavioral variables (i.e., violations, lapses and errors) which were able to predict accident risk. Specifically, these studies suggested that this theoretical model is effective in predicting risky driving behaviors in different categories of drivers. It is important to note that the associations within the variables located at the same level in the model (i.e., personality level and behavioral level) were consistent across the different groups of drivers (i.e., Ulleberg and Rundmo, 2003; Lucidi et al., 2010, 2014, 2019; Mallia et al., 2015). However, the results of the studies cited above, also showed there may be variations in the pattern of relationships between the variables depending on the types of the driver. These variations could be at a distal level (i.e., effects of personality traits on attitudes), at a proximal level (i.e., effects of attitudes on behavioral level), or in between (i.e., effects of personality traits on behavior) and also that they could be partly explained by the peculiarities of the driver’s groups so that, for example, the effects (direct or indirect) of the personality characteristics on drivers’ behaviors could differ across drivers ages and drivers experience groups. Additionally, these variations could also depend on the differences in the driving behaviors addressed in the studies. For example, Ulleberg and Rundmo (2003) examined self-assertiveness and rule violations, whereas more recent studies (Lucidi et al., 2014; Mallia et al., 2015) analyzed violations, errors, and lapses. In order to further investigate these disparities, direct comparisons of different groups of drivers through multi-group analyses are needed. Previous studies have demonstrated that age is a relevant factor on which to make comparisons across groups. The results of different meta-analyses (de Winter and Dodou, 2010; de Winter et al., 2015) showed not only that driving behaviors varied across ages, but also that the effects of the latter on accident involvement do. For the first set of differences, we know for example that younger drivers commit more violations and errors than older drivers. Secondly, results showed that violations are a stronger predictor of accidents amongst young drivers than they are amongst older drivers. Furthermore, although positive attitudes toward traffic safety improve with age (e.g., Iversen and Rundmo, 2004; Nordfjærn et al., 2010), age did not moderate the effects of attitudes on driving behaviors. Many studies have found that attitudes toward traffic safety predicted mainly volitional behavior (e.g., violations and errors), whereas to a lesser extent they predict behaviors that are not under the control of the individual (e.g., lapses) (e.g., Lucidi et al., 2014; Mallia et al., 2015). This happens to the same degree for different drivers’ age groups. Lastly, with regards to personality traits, previous findings showed that, for example, the average level of excitement-seeking decreased with age (Steinberg et al., 2008; Nordfjærn et al., 2010); nevertheless, age did not moderate the effects of excitement-seeking on driving behaviors (e.g., Lucidi et al., 2014; Mallia et al., 2015). In fact, excitement seeking affected violations in all the tree age groups of drivers to the same amount.

Although previous research has analyzed differences in drivers from different age groups at different levels, none of them has compared the overall model in a large sample of drivers from different ages. This study is the first to investigate the ‘personality–attitudes–risky driving behavior’ model of Ulleberg and Rundmo (2003) across different age groups of drivers. A comparison of drivers from different age group could explain disparities in how personality characteristics and attitudes toward traffic safety predict risky driving behavior and accident risk.

### The Study Aims and Hypothesis

The purposes of this paper are threefold. First, through a multigroup analysis, we want to establish the measurement invariance of the ‘personality–attitudes–risky driving behavior’ model of Ulleberg and Rundmo (2003) in order to predict risky driving behaviors, including accidents risk and issued traffic fines, (e.g., Lucidi et al., 2014; Mallia et al., 2015) across different age groups. Subsequently, we intend to evaluate the differences between age groups concerning the impact of personality characteristics and attitudes on aberrant driving behavior and car accidents/traffic fines. The present study is the first to analyze the model measurement invariance in different age groups and then comparing effects across ages. In addition, this is the first study conducted on a large sample of drivers who were representative of the Italian population: this was particularly valuable for the estimation of group differences. The study answers the following research questions:

(1) Does the model globally predict risky driving behavior and accident risk/fines received in different age groups of drivers?
(2) What are the differences between young, adult and older drivers in the impact of personality traits and attitudes on driving behaviors and accident risk/fines received?

## Materials and Methods

### Sample

Overall, the study involved 1,286 Italian active drivers from three different age groups, namely young (*n* = 435, mean age = 18.43 years; *SD* = 0.63), adult (*n* = 412, mean age = 40.61; *SD* = 8.90) and older drivers (*n* = 439 = mean age = 68.91 years; *SD* = 5.95). Participants from all the age groups were recruited separately through a convenience sampling procedure by trained associate researchers of Sapienza University of Rome. The criterion for selection was: younger than 20 years, between 25–59 years and 60 years or older for young, adult and older driver, respectively. The study was approved by the Institutional Review Board of the Department of Social and Developmental Psychology of La Sapienza University of Rome. Following international standards for ethics in behavioral research, all participants were informed about the study and required to give their consent for participation. All of the contacted participants agreed to take part in the study, gave their written informed consent and completed a booklet of structured and validated anonymous questionnaires, which lasted approximately 30 min. All the participants had a valid driving license and were active drivers.

### Measures

#### Questionnaires Assessing Drivers’ Personality

Drivers’ personality traits were assessed using four facets of the Italian version of the “NEO Personality Inventory-Revised” (NEO-PI-R; Costa and McCrae, 1992; Caprara et al., 2001): (a) excitement-seeking (E5; e.g., “I often crave excitement”); (b) angry hostility (N2; e.g., “I often get angry at the way people treat me”); (c) anxiety (N1; e.g., “I often feel tense and jittery”); and (d) altruism (A3; e.g., “I generally try to be thoughtful and considerate”). There were eight items for each facet, with responses given on a five-point Likert-type scale ranging from strongly disagree (1) to strongly agree (5) endpoints. Past studies have ascertained a good internal reliability for this measure in Italian (α ranging from 0.85 to 0.91), American (α ranging from 0.89 to 0.95) and Norwegian (ranging from 0.75 to 0.80) samples (Caprara et al., 2001; Källmen et al., 2011) as well as evidences about its factorial, convergent and divergent validity (see Costa and McCrae, 1992).

Drivers’ normlessness (i.e., the belief that socially unapproved behaviors are required to achieve certain goals) was evaluated using the “Normlessness Scale” by Kohn and Schooler (1983). The scale comprises four items (e.g., “If something works, it is less important whether it is right or wrong”) with responses given on five-point Likert-type scales ranging from strongly disagree (1) to strongly agree (5). Past studies reported acceptable reliability coefficients for this measure in drivers from different countries such as Norway (α = 0.71) (Ulleberg and Rundmo, 2003), China (α = 0.63) (Yang et al., 2013) and Italy (α ranging from 0.61, in older drivers, to 0.72 in professional drivers; Lucidi et al., 2010, 2014; Mallia et al., 2015).

#### Questionnaire Assessing Drivers’ Attitudes Toward Traffic Safety

Drivers’ attitudes toward road-safety were assessed through the Italian version of the 11-item scale originally developed by Iversen and Rundmo (2004), Lucidi et al. (2010, 2014), Mallia et al. (2015). Specifically, the scale measures attitudes toward rule violations and speeding, higher scores on this scale reflect positive attitudes toward traffic safety (e.g., “Traffic rules must be respected regardless of road and weather conditions”). For each item, participants were asked to indicate their level of agreement on a five-point Likert-type scale ranging from strongly disagree (1) to strongly agree (5).

#### Questionnaire Assessing Drivers’ Behaviors

Drivers’ behavior at the wheel was measured using the Italian version of the 28-item Driver Behavior Questionnaire (DBQ, Lawton et al., 1997; Lucidi et al., 2010, 2014; Mallia et al., 2015). Participants were asked to rate how often, in the last year, they committed specific driving violations (12 items, e.g., “Disregard the speed limit on a residential road”), errors (8 items, e.g., “Underestimate the speed of an oncoming vehicle when overtaking”), and lapses (8 items, e.g., “Misread the signs and exit from a roundabout on the wrong road”), with responses given on a six-point Likert-type scale from never (0) to nearly all the time (5).

#### Assessment of Crash Involvement and Traffic Law Violations

Finally, participants were asked to indicate whether they received fines (Yes/No) and they were involved in car crash as drivers with property damage and/or with physical injury (Yes/No) in the last year.

### Data Analysis

First of all, for all the key variables of the study differences across the three groups were explored trough ANOVAs and chi-square tests using IBM SPSS Statistics, version 25 (IBM Corp, 2017). Then, fit of the Rundmo and Ulleberg’s ‘personality–attitudes–risky driving behavior’ model with the data was tested using variance-based structural equation modeling (VB-SEM – also known as Partial Least Squares analysis) with the WARP PLS v.6.0 statistical software (Kock, 2017). More specifically, a multigroup analysis (Kock, 2014) was carried out in order to evaluate the model measurement invariance parameters across the three samples of drivers and the extent to which the model’s hypothesized relations held across them.

In order to calculate the measurement indicators for all the key latent variables of the tested model (i.e., anxiety, hostility, excitement-seeking, altruism, normlessness, positive attitudes, violations, lapses and errors), an item parceling procedure^1^ (Kim and Hagtevt, 2003) was used in line with previous studies (e.g., Lucidi et al., 2014; Mallia et al., 2015). Specifically, the item parcels for each latent variable (i.e., anxiety, hostility, excitement-seeking, altruism, normlessness, attitudes, violations, lapses, and errors) were created by randomly grouping the items of each scale into three separate item sets (parcels) and by averaging the item scores within each set. The dichotomous variables (Yes/No) about crash involvement and fines received were considered observed measures in the tested model.

Measurement-level statistics of the VB-SEM of the model data were firstly examined to ensure whether the latent variables met construct and discriminant validity criteria. Construct validity of the latent factors was tested for each factor using average variance extracted (AVE) and composite reliability coefficient (ρ), which should exceed 0.50 and 0.70, respectively. On the other hand, adequacy of the hypothesized model was established for each sample using an overall goodness-of-fit (GoF) index given by the square root of the product of the AVE and average *R*^2^ for the model, with values of 0.100, 0.250, and 0.360 corresponding to small, medium, and large effect sizes, respectively (Tenenhaus et al., 2005). Further information on the adequacy of the models was provided by the average path coefficient (APC) and average *R*^2^ coefficient (ARS) across the model parameters, both of which should be statistically significantly different from zero. With respect to the model effects, each structural relation among model constructs was estimated using standardized coefficients, and test of difference from zero.

## Results

### The Key Variables of the Study Across the Three Age Groups of Drivers

Table 1 shows participants’ socio-demographic characteristics and driving habits for each age groups. The three groups were significantly different obviously for age [*F*_(2,1283)_ = 7444.15; *p* < 0.001] and for the number of years they have held a full driving license for [*F*_(2,1283)_ = 3640.43; *p* < 0.001]. The three groups differed also for gender composition [*X*^2^_(2)_ = 32.17; *p* < 0.001], being the young and older drivers predominantly males (61.4% and 62.9%, respectively) compared to adult drivers (45.4%). With respect to driving tendencies, 74.0% of adults and 60.2%, of older participants reported daily driving, whereas only about 40% of the young participants drove every day [*X*^2^_(2)_ = 108.57; *p* < 0.001]. On the other hand, older drivers reporting to drive for more than 100 km per week were significantly fewer than young and adult driving for so long distance [*X*^2^_(2)_ = 28.43; *p* < 0.001; older: 33.2%; adult: 48.3%; young: 49.3%]. Furthermore, adult drivers significantly drove more frequently for more than 2 h with respect to young and older drivers [*X*^2^_(2)_ = 16.94; *p* < 0.001; adult: 17.2%; young: 9.9%; older: 8.8%]. Contrarily, a statistically significant higher proportion of young drivers reported to drive at night time, compared to older drivers [*X*^2^_(2)_ = 127.411; *p* < 0.001; 32.9%, 3.2%, respectively].

**Table 1:** Socio-demographic characteristics and driving habits for the three groups of drivers.

	Young drivers (*n* = 435)	Adult drivers (*n* = 412)	Older drivers (*n* = 439)
Age range	18–24 years old	25–59 years old	61–90 years old
Mean age (*SD*)	18.43 (.63)	40.61 (8.90)	68.91 (5.95)
% Male	61.4% ^B^	45.4% ^A,C^	62.9%^B^
Mean years that they have driver’s license	0.58 (.48) ^B,C^	20.15 (8.90) ^A,C^	44.01 (8.91)^A,B^
Driving every day	38.9% ^B^	74.0% ^A^	60.6%
Driving more than 100 km per week	49.3% ^C^	48.3%^C^	33.2% ^A,B^
Prolonged driving (more than 2 h) more than four times per month	9.9% ^B^	17.2% ^A,C^	8.8% ^B^
Driving at night more than four times per month	32.9% ^C^	19.2%	3.2% ^A^

*^*A*-*C*^ Age related drivers’ groups resulting significantly different at Bonferroni *post hoc* test (*p* < 0.001) or Haberman’ analysis of standardized residuals.*

Overall, the three groups showed statistically significant differences also with respect to personality traits [anxiety: *F*_(2,1283)_ = 26.12, *p* < 0.001; hostility: *F*_(2,1283)_ = 26.75, *p* < 0.001; excitement -seeking: *F*_(2,1283)_ = 220.08, *p* < 0.001; altruism: *F*_(2,1283)_ = 15.58, *p* < 0.001; normlessness: *F*_(2,1283)_ = 20.23, *p* < 0.001], positive attitudes toward traffic safety [*F*_(2,1283)_ = 36.34, *p* < 0.001] and self-reported aberrant driving behaviors [violation: *F*_(2,1283)_ = 97.56, *p* < 0.001; lapses: *F*_(2,1283)_ = 16.55, *p* < 0.001; errors: *F*_(2,1283)_ = 40.56, *p* < 0.00]. In particular, as reported in Table 2, the *post hoc* comparisons revealed that for all the key variables of the study, there was at least one difference between the three groups, with the young drivers reporting the highest values in anxiety, hostility, excitement seeking, and errors and the lowest values in altruism, normlessness and positive attitudes toward traffic safety. Conversely, older drivers showed the lowest scores in hostility, excitement-seeking, violations, lapses and errors, and the highest scores in altruism and positive attitudes toward traffic safety. Finally, adult drivers showed a median position reporting significantly different scores from young and older drivers in hostility, excitement-seeking, positive attitudes toward traffic safety, and errors.

**Table 2:** Measurement model statistics and factor intercorrelations for latent variables in the three samples.

	Age group	Mean/%	*SD*	ρ	AVE	1	2	3	4	5	6	7	8	9	10	11
1. Anxiety	Young Adult Older	3.20^B,C^ 2.89^A^ 2.93^A^	0.63 0.70 0.70	0.84 0.86 0.83	0.63 0.68 0.63	0.79 0.82 0.79										
2. Hostility	Young Adult Older	2.87 ^B,C^ 2.73 ^A,C^ 2.56 ^A,B^	0.62 0.66 0.60	0.81 0.82 0.79	0.59 0.60 0.55	0.40^∗∗∗^ 0.40^∗∗∗^ 0.36^∗∗∗^	0.77 0.78 0.75									
3. Excitement seeking	Young Adult Older	3.23 ^B,C^ 2.77 ^A,C^ 2.22 ^A,B^	0.71 0.77 0.63	0.83 0.84 0.78	0.63 0.63 0.54	-0.16^∗^ -0.12^∗^ -0.17^∗∗∗^	0.13^∗∗^ 0.20^∗∗∗^ 0.17^∗∗∗^	0.79 0.80 0.73								
4. Altruism	Young Adult Older	3.83 ^B,C^ 3.94 ^A,C^ 4.04 ^A,B^	0.58 0.54 0.56	0.84 0.84 0.83	0.63 0.63 0.62	-0.03 -0.04 0.05	-0.32^∗∗∗^ -0.35^∗∗∗^ -0.30^∗∗∗^	0.02 -0.20^∗∗∗^ -0.10^∗^	0.79 0.80 0.78							
5. Normlessness	Young Adult Older	2.24 ^B,C^ 2.51 ^A^ 2.44 ^A^	0.73 0.58 0.58	0.81 0.86 0.84	0.58 0.68 0.65	-0.08 0.02 0.04	0.17^∗∗∗^ 0.23^∗∗∗^ 0.09	0.37^∗∗∗^ 0.39^∗∗∗^ 0.21^∗∗∗^	-0.13^∗∗^ -0.16^∗∗^ 0.01	0.76 0.82 0.80						
6. Attitudes	Young Adult Older	3.41 ^B,C^ 3.57 ^A,C^ 3.80 ^A,B^	0.63 0.71 0.69	0.85 0.88 0.87	0.64 0.70 0.70	0.08 0.09 0.06	-0.16^∗∗∗^ -0.13^∗∗^ -0.23^∗∗∗^	-0.31^∗∗∗^ -0.39^∗∗∗^ -0.22^∗∗∗^	0.22^∗∗∗^ 0.22^∗∗∗^ 0.13^∗∗^	-0.52^∗∗∗^ -0.47^∗∗∗^ -0.32^∗∗∗^	0.80 0.84 0.84					
7. Violations	Young Adult Older	1.22 ^C^ 1.26 ^C^ 0.65 ^A,B^	0.78 0.84 0.52	0.91 0.94 0.90	0.78 0.84 0.76	-0.13^∗∗^ -0.12^∗^ 0.04	0.26^∗∗∗^ 0.28^∗∗∗^ 0.29^∗∗∗^	0.41^∗∗∗^ 0.48^∗∗∗^ 0.28^∗∗∗^	-0.22^∗∗∗^ -0.29^∗∗∗^ -0.18^∗∗∗^	0.43^∗∗∗^ 0.41^∗∗∗^ 0.22^∗∗∗^	-0.49^∗∗∗^ -0.46^∗∗∗^ -0.45^∗∗∗^	0.88 0.92 0.87				
8. Lapses	Young Adult Older	0.98 ^C^ 0.89 ^C^ 0.73 ^A,B^	0.68 0.62 0.60	0.86 0.88 0.89	0.68 0.70 0.73	0.16^∗∗∗^ 0.14^∗∗^ 0.16^∗∗∗^	0.25^∗∗∗^ 0.17^∗∗∗^ 0.29^∗∗∗^	0.13^∗∗^ 0.04 0.07	-0.24^∗∗∗^ -0.02 -0.13^∗∗^	0.19^∗∗∗^ 0.21^∗∗∗^ 0.10^∗^	-0.24^∗∗∗^ -0.06 -0.17^∗∗∗^	0.38^∗∗∗^ 0.29^∗∗∗^ 0.51^∗∗∗^	0.82 0.84 0.86			
9. Errors	Young Adult Older	0.70 ^B,C^ 0.54 ^A,C^ 0.38 ^A,B^	0.58 0.52 0.44	0.87 0.89 0.89	0.69 0.73 0.72	0.01 0.03 0.08	0.19^∗∗∗^ 0.16^∗∗∗^ 0.23^∗∗∗^	0.24^∗∗∗^ 0.28^∗∗∗^ 0.11^∗^	-0.28^∗∗∗^ -0.19^∗∗∗^ -0.14^∗∗^	0.26^∗∗∗^ 0.35^∗∗∗^ 0.18^∗∗∗^	-0.28^∗∗∗^ -0.29^∗∗∗^ -0.22^∗∗∗^	0.55^∗∗∗^ 0.56^∗∗∗^ 0.57^∗∗∗^	0.56^∗∗∗^ 0.54^∗∗∗^ 0.68^∗∗∗^	0.83 0.85 0.85		
10. Involvement in at least one accident (Yes/No)	Young Adult Older	20.7% ^C^ 10.3% ^A^ 7.6% ^A^	– – –	1.00 1.00 1.00	1.00 1.00 1.00	-0.04 0.01 0.00	0.12^∗^ 0.07 -0.04	0.12^∗^ 0.03 0.08	-0.08 -0.03 0.01	-0.13^∗∗^ -0.00 0.08	-0.18^∗∗∗^ -0.09 0.00	0.28^∗∗∗^ 0.19^∗∗∗^ 0.07	0.22^∗∗∗^ 0.10^∗^ 0.04	0.21^∗∗∗^ 0.19^∗∗∗^ 0.11^∗^	1.00 1.00 1.00	
11. Received at least one fine (Yes/No)	Young Adult Older	14.6% ^B^ 39.8% ^A^ 26.7%	– – –	1.00 1.00 1.00	1.00 1.00 1.00	-0.01 -0.04 -0.06	-0.00 0.07 0.08	0.14^∗∗^ 0.15^∗∗^ 0.13^∗∗^	-0.02 -0.05 -0.10^∗^	0.06 0.14^∗∗^ 0.05	-0.17^∗∗∗^ -0.12^∗^ -0.17^∗∗∗^	0.24^∗∗∗^ 0.24^∗∗∗^ 0.27^∗∗∗^	0.09 0.05 0.11^∗^	0.12^∗^ 0.12^∗^ 0.16^∗∗∗^	0.22^∗∗∗^ 0.12^∗^ 0.02	1.00 1.00 1.00

*Coefficients on the upper line are for young drivers (A), coefficients on the middle line are for adult drivers (B), and coefficients on the lower line are for older drivers (C). ^*A*-*C*^ Drivers’ groups resulting significantly different at Bonferroni *post hoc* test (*p* < 0.001) or Haberman’ analysis of standardized residuals. ρ, composite reliability coefficient; AVE, average variance extracted; values on principal diagonal are squared-root of AVE. ^∗∗∗^*p* < 0.001; ^∗∗^*p* < 0.01; ^∗^*p* < 0.05.*

Finally, a statistically significant higher percentage of young drivers reported to be involved in a car accident in the last year compared to adult and older drivers [*X*^2^_(2)_ = 36.58; *p* < 0.001; young: 20.7%; adult: 10.3%; older: 7.6%]. Furthermore, higher rates of adult drivers received at least one fine in the last year, with respect to young drivers [*X*^2^_(2)_ = 72.25; *p* < 0.001; 39.8% and 14.6, respectively], whereas no significant differences emerged in fines received in older drivers.

### The Construct Validity of the Scales and Measurement Invariance of the Personality–Attitudes–Behavior Model

**FIGURE 1:**
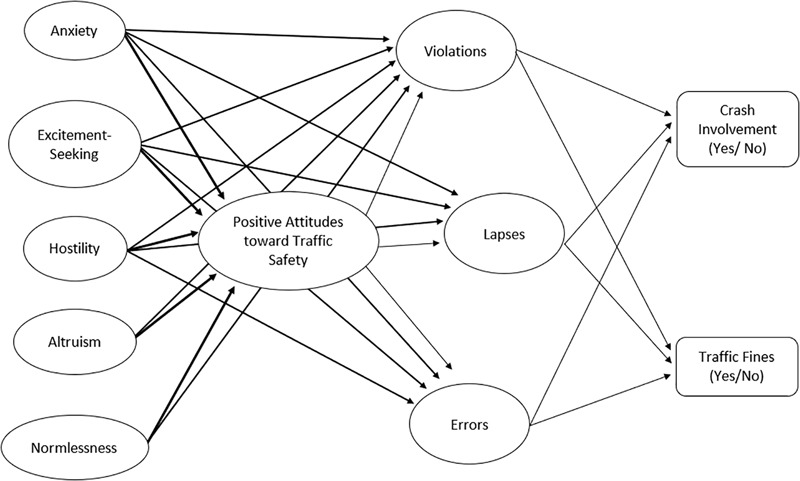
The personality–attitude–behavior model tested in the three samples (i.e., young, adult, and older) of drivers.

Composite reliability coefficients, AVE for the factors, and factor intercorrelations are presented in Table 2. Overall, reliability coefficients exceeded the 0.700 criterion for all the factors included in the model and for each of the three samples analyzed. Furthermore, in all cases the square root of the AVE for each latent variable exceeded the correlation between all the variables. With respect to the adequacy of the hypothesized model (Figure 1), the results showed that it exhibited a good fit with the data collected in young (GoF = 0.367; APC = 0.150, *p* < 0.001; ARS = 0.189, *p* < 0.001; AFVIF = 1.497), adult (GoF = 0.362; APC = 0.140, *p* < 0.001; ARS = 0.176, *p* < 0.001; AFVIF = 1.497) and older drivers (GoF = 0.310; APC = 0.124, *p* = 0.002; ARS = 0.134, *p* = 0.001; AFVIF = 1.463). The multigroup analysis on the measurement level of the model provided support for the measurement invariance^2^, showing that factor loadings for each variable considered in the model were statistically equivalent across the three samples, and thus allowing us to meaningfully compare the model’s paths of the three age groups of drivers.

### Differences in the Model’s Paths According to Age

Differences in the model standardized path coefficients across the three age groups are shown in Table 3.

**Table 3:** Model paths estimations and comparisons across the three samples of drivers.

Model paths	Samples		
	Young ^(A)^	Adults ^(B)^	Older ^(C)^
Anxiety → Attitudes	0.048	0.078	0.097*
Hostility → Attitudes	-0.050C	-0.025C	-0.190***A,B
Excitement Seeking→ Attitudes	-0.149***	-0.225***C	-0.099**^B^
Altruism → Attitudes	0.167***C	0.137**C	0.023 ^A,B^
Normlessness → Attitudes	-0.429***C	-0.379***	-0.289***^A^
Anxiety → Violations	-0.126**C	-0.139**C	0.026 ^A,B^
Hostility → Violations	0.172***	0.203***	0.156***
Excitement Seeking → Violations	0.235***	0.282***	0.177***
Altruism → Violations	-0.100*	-0.119**	-0.062
Normlessness → Violations	0.132**	0.116**	0.062
Anxiety → Lapses	0.153***	0.068	0.143**
Hostility → Lapses	0.150***	0.206***	0.207***
Excitement Seeking→ Lapses	0.112**	0.011	0.073
Anxiety → Errors	-0.039 ^B,C^	0.100*A	0.102*A
Hostility → Errors	0.129**	0.132**	0.156***
Excitement Seeking→ Errors	0.169***C	0.203***C	0.044 ^A,B^
Attitudes→ Violations	-0.296***	-0.229***C	-0.348***^B^
Attitudes→ Lapses	-0.230***B	-0.060 ^A^	-0.183***
Attitudes→ Errors	-0.209***	-0.202***	-0.128**
Violations →Fines	0.253***	0.250***	0.278***
Lapses →Fines	0.002	-0.008	0.042
Errors →Fines	-0.017	-0.019	0.041
Violations → Accidents	0.200***C	0.134**C	0.005 ^A,B^
Lapses → Accidents	0.113**B,C	0.055 ^A^	-0.011A
Errors → Accidents	0.079**	0.115**0	0.154***

*^*A*-*C*^ Drivers resulted significantly different for *p* < 0.05 (one-tailed) using pooled standard error method. ^∗∗∗^*p* < 0.001; ^∗∗^*p* < 0.01; ^∗^*p* < 0.05.*

A first set of differences was related to the model’s paths linking drivers’ personality traits to positive attitudes toward traffic safety. Specifically, anxiety and hostility have statistically significant (positive and negative, respectively) relationships with drivers attitudes only in older drivers. Contrarily, excitement-seeking and normlessness showed statistically significant and negative relationships with attitudes in all the three samples, although the relationship between excitement seeking and attitudes resulted significantly stronger in adult than in older drivers, and the relationship between normlessness and attitudes was significantly stronger in young than in older drivers. Finally, altruism significantly and positively affected only young and adult attitudes, and not older’ ones.

A second set of differences concerns the direct effects of drivers’ personality traits on violations, lapses and errors. In particular, anxiety and altruism (negatively) and normlessness (positively) were significantly associated with violations only in young and adult drivers. Anxiety resulted significantly and positively related to driver lapses only in young and older drivers, whereas excitement seeking was associated with lapses only in young drivers. Furthermore, anxiety had a positive association with drivers’ errors, both in adult and older drivers, whereas excitement seeking was associated with errors in adult and young drivers. Finally, hostility had a statistically significant and positive impact on all the three categories of aberrant behaviors at the same level across the samples.

The third set of differences deals with the extent to which drivers’ positive attitudes toward traffic safety were associated with risky driving behaviors. The results showed that attitudes significantly and negatively affected violations in all the three groups of drivers, although this relationship resulted significantly stronger in older than in adult drivers. Furthermore, the results showed that attitudes significantly and negatively affected lapses only in young and older drivers. Conversely, errors were significantly and negatively related to attitudes at the same level across the three samples.

Finally, the fourth set of differences was related to the capacity of the model to predict fines and car accidents involvement. With respect to fines, the results showed no statistically significant differences across the three groups, being the violations the only risky behavior that was positively related to past fines. In contrast, with respect to accidents involvement, violations seemed to have a positive impact for young and adult but not for older drivers, whereas lapses affected accident involvement only for young drivers. Finally, errors resulted positively associated with car accidents involvement in all the three groups of drivers.

## Discussion

The relationship between personality, attitudes and risky driving behaviors has been systematically evaluated through the Ulleberg and Rundmo’s (2003) model, with the inclusion of the prediction of car accidents risk and issued traffic fines (e.g., Lucidi et al., 2014; Mallia et al., 2015). Previous studies tested the model in different samples of drivers (e.g., Ulleberg and Rundmo, 2003; Lucidi et al., 2014; Mallia et al., 2015), but none of them so far had established the measurement invariance of the model across different age groups. Moreover, neither study analyzed the differences in the effects across young, adult and older drivers. Because there is evidence that personality characteristics and prosafety attitudes can affect risky driving behavior and car accidents differently depending on the age and experience of the drivers (e.g., Ulleberg and Rundmo, 2003; Lucidi et al., 2014; Mallia et al., 2015), in the present study, we analyzed these specific divergences adopting Ulleberg and Rundmo’s model in a representative sample of Italian drivers. In order to have a reliable estimation of the differences between the different samples of drivers, we first assessed the measurement invariance of the model across the three age groups. The results showed that, in the three samples of drivers, the measurement model fitted the data well, revealing the adequacy of the model measurement in the different age groups. This is in line with previous studies conducted using this model (Lucidi et al., 2014; Mallia et al., 2015). Furthermore, the model showed measurement invariance across age groups, meaning that young, adult and older drivers appear to conceptualize the model factors and to interpret the corresponding items in similar way. This provided us with a relevant estimation of the differences in the model’s paths between the various age groups of drivers. Differences emerged at several levels in the model.

At a distal level (i.e., linking personality traits with attitudes toward traffic safety), anxiety and hostility predicted drivers’ attitudes toward traffic safety (positively and negatively, respectively) only in older drivers. Excitement-seeking and normlessness predicted attitudes in all the three samples, even if to a different extent depending on the age. Finally, altruism affected positive attitudes toward traffic safety significantly and positively only in young and adults and not in older drivers. This result is in line with previous studies on drivers of different ages (Ulleberg and Rundmo, 2003; Lucidi et al., 2014; Nordfjærn et al., 2015; Zicat et al., 2018), on professional drivers (e.g., Mallia et al., 2015), and on motorcycle drivers (Chen, 2009).

At a more proximal level (i.e., linking drivers’ attitudes with risky behaviors at the wheel), differences were slightly lessened. In fact, although attitudes didn’t impact lapses significantly in older drivers and they impacted violation more relevantly in older than in adult drivers, positive attitudes toward traffic safety were globally related to less violations and errors in the three age groups. This confirms the universal and favorable impact of positive attitudes toward traffic safety on risky driving behaviors (Lucidi et al., 2014; Mallia et al., 2015).

Considering the effects of personality characteristics on risky driving behaviors at the wheel, the results showed that excitement seeking affected violations in all the tree age groups of drivers to the same extent. This is in line with previous research conducted in drivers of different ages, showing that this personality dimension has a central role in deliberate actions, which deviates from rules or safe driving practices (e.g., ***Schwebel et al., 2007***; Nordfjærn et al., 2015; ***Shen et al., 2018***). On the other hand, results suggested that excitement seeking had an impact on both errors and lapses only in young drivers, and only on errors in adult drivers. This is in accordance with previous studies on older drivers showing that sensation-seeking doesn’t have a role in determining non-volitional aberrant driving behaviors in older people (e.g., Lucidi et al., 2014). Differences among the three age groups emerged also with respects to some specific personality traits related to emotionality and their link to risky driving behavior. For example, higher levels of anxiety were linked with more self-reported lapses in young and older drivers, and with more self-reported errors in adult and older drivers. This is in line with the finding that anxiety is related to increased reaction times on tasks and also with increased errors and lapses (Pourabdian and Azmoon, 2013; Wong et al., 2015). Past evidences also addressed that drivers with an anxious driving style showed lapses in attention and memory (Lucidi et al., 2010), a higher number of errors while driving (Taylor et al., 2007), and they are more likely to be involved in car crashes (Marengo et al., 2012). Conversely, the results of the present study showed that higher scores in anxiety resulted in fewer self-reported violations in young and adult drivers, confirming past studies on novice young drivers (Lucidi et al., 2010), and supporting the hypothesis that in young and adult drivers, anxiety may stimulate doubts about their driving abilities, or may induce accidents’ fear, leading them to be more cautious, and consequently, to report less violations. However, the research about the exact role of anxiety on risky driving is very limited and additional research on this topic are needed. For instance, future studies could investigate the effect of anxiety on risky driving behavior by longitudinal designs (Barnard and Chapman, 2018). In the opposite way, higher scores in hostility resulted in higher frequency of self-reported traffic rule violations, lapses and errors in all the three age groups. This is consistent with previous studies on drivers of different ages showing that hostility can be ex***h***ibited through observable actions, like for example offensive hand gestures, honking the horn, and even through aggressive behaviors toward other drivers, that distract the drivers from their driving, increasing the probability to commit errors, to miss some crucial information (i.e., lapses) or to deliberately break traffic rules (e.g., Beanland et al., 2014; Gidron et al., 2014; Zhang et al., 2015).

Finally, at the behavioral level, differences emerged also in the link between aberrant self-reported driving behaviors (lapses, errors, and violations) and the more objective measures of risky driving, that are crash involvement and fines. Although the results showed that a higher number of self-reported violations were related to a greater likelihood to receive traffic fines, and errors had a key role for the risk to be involved in a car accident in all the three samples, violations were associated with higher risk of car accidents only in young and adult drivers, and, lapses were related to car accidents only in young drivers. Overall, the findings of this study are consistent with previous research focused on samples of drivers of different ages (de Winter and Dodou, 2010; de Winter et al., 2015; Martinussen et al., 2014, 2017) and attesting that the aberrant behaviors at the wheel influence the accident risk differently across the life-span. In fact, according to our results, for example, committing a violation resulted in a higher accident risk in young and adult drivers, whereas older drivers committing violations didn’t have this consequence. This is probably due the fact that young and adult drivers may commit more dangerous violations (e.g., do not stop at the red light) that seriously impact traffic safety, compared to older drivers, who commit violations with less impact on driving safety (e.g., honk the horn to express annoyance). Similarly, driving lapses due to lack of attention and/or memory (e.g., “Hit something when reversing that you had not previously seen”) seemed to increase the likelihood to have an accident only in young drivers, probably due to a lack of experience in these drivers that globally increases the accident risk. Conversely, with the experience, adult and older drivers learn to manage these inattentions with corrective or preventive maneuvers (e.g., “look at the rearview mirror twice”), counterbalancing the possibility to lead to an accident.

The most original addition of this study was to establish the measurement invariance of a comprehensive model that predicts risky driving behaviors: the ‘personality–attitudes–risky driving behavior’ model of Ulleberg and Rundmo (2003), recently extended in order to include the prediction of accidents risk and issued traffic fines (e.g., Lucidi et al., 2014; Mallia et al., 2015) across different age groups of drivers. The present study is the first to analyze the model measurement invariance of Ulleberg and Rundmo (2003) across age groups, confirming the applicability and the universality of this model across age groups. This is important as firstly, it demonstrates that, even at different age and driving experience, drivers interpret the key variables included in the model in a conceptually similar manner. Secondly, it confirms that the model is able to predict aberrant behavior at the wheel and car accidents/traffic received in the three age groups, so that some specific aspects related to age, such as changes in cognitive abilities, do not seem to impact the applicability of the model.

The present study is also the first to analyze differences across the groups at different level in the model. Findings of multigroup analysis showed that differences between the samples emerged at all levels in the model – at the link of personality traits with attitudes, attitudes with driving behavior at the wheel and lastly, driving behavior at the wheel with car accident risk and issued fines.

### Practical Implications

The results of the present study have different implications. First of all, they confirm the key role of attitudes toward traffic safety in predicting violations, lapses and errors and in mediating the effect of personality on these risky driving behaviors on young, adult and older drivers, suggesting to focus the interventions upon drivers’ attitudes. As attitudes are not stable as personality traits are, interventions addressing attitudes can produce long-term changes in drivers directly affecting risky behavior (e.g., Albarracin and Shavitt, 2018). In particular, as pointed out by Goldenbeld et al. (2000), the focus on drivers attitudes is crucial for risky behaviors that are under the control of the drivers; for example, Rothengatter and Manstead (1997) demonstrated that attitudes were relevant for speed choice. Furthermore, as we know that the attitude–behavior relationship is not generalized (Goldenbeld et al., 2000), situation-specific programs, which also consider the specific behavior under study, could be more efficacious in reducing risky driving in different target groups. However, influencing attitudes though educational intervention has also several limitations, as specified by Goldenbeld et al. (2000). Another strategy would be to focus the interventions upon the emotional factors, such as anxiety and hostility, that in the present study resulted directly related to aberrant driving behaviors across ages. In fact, lapses or errors at the wheel may be caused by drivers’ emotional states (Wong et al., 2015). Different studies in the behavioral sciences and neuroscience have shown that poor emotion and self-regulation are also associated with a wide range of risk-taking and health compromising behaviors especially among young people (e.g., Magar et al., 2008). Similarly, hostility expressed in driving situations may trigger also aggressive behavioral responses that distracted drivers from driving and increase the probability to commit violations, errors or lapses, thus putting drivers and passengers’ security at risk. The interventions designed to promote traffic safety in drivers of different ages, thus, should work on including emotion regulation strategies, specifically in traffic situations. In fact, recent empirical evidences (e.g., Šeibokaitë et al., 2017) showed difficulties in emotion regulation are significantly related with driving errors, lapses, aggressive violations, and ordinary violations in drivers of different ages. In this regard, experimental studies have demonstrated the effectiveness of cognitive, relaxation, and behavioral interventions – and their combinations – in reducing driving anger and aggression in angry drivers (for a review, see Deffenbacher, 2016). Last, it is important to note that the results of the present study also allow tailoring the interventions to the specific target group. For example, while with young and adult drivers’ actions should be made to promote the emotional regulation in complex traffic situations or in reducing driving anger and aggression in angry drivers, the problems with older drivers is that of reducing anxiety which could lead to more lapses and errors.

Aside from the all above considerations, we must note that the prevention of crash rates and the promotion of safe driving do not depend only on drivers’ behaviors and attitudes but strong efforts should be rather made by government policy makers in order to assure the development of safe infrastructures.

### Limitations

One of the limitations of the study is that it used a cross-sectional design that has limited the possibility to establish any causality in the relationships observed within the model. However, personality traits are known to be stable over years and to predict behaviors (Fishbein, 2009), so this may have reduced the possibility that attitudes and driving behaviors could have preceded and influenced the personality. Secondly, since different age groups were compared at the same time with a cross-sectional design, there is the possibility that the effects we observed are due to the effect of age (i.e., maturational effect) but also to the specific cohort to which they belong. Only longitudinal designs may help, in future study, to understand clearly the unique contribution of each possible effect. A third limitation is the use of self-reported measures to assess driving behavior and this may have exposed to report or recall biases. However, a meta-analysis conducted by de Winter and Dodou (2010) demonstrated that the questionnaire used in the present study, was a valid predictor of car accident involvement. A fourth limitation is that the present study is based on Italian drivers, so the sample is not representative of the worldwide population. Despite these limitations, present results support the applicability of the model in different age groups of drivers and show the different effects of personality characteristics and attitudes on aberrant behaviors at the wheel and on crash risk/fines received between young, adult and older drivers.

## Conclusion

The measurement invariance of the model tested in the current study is useful as it provides a valid tool to compare model relationships across different groups of drivers depending on the age. The present study showed also differences between young, adult and older people in the model relationships, highlighting some peculiarities in the way personality traits and attitudes affects risky driving behavior and car accident/issued fines due to age and driving experience. An extension of the present study would be to investigate the differences in the model relationships between groups of drivers who differ each other for other personal characteristics, such as gender or cultural background.

## Data Availability

The datasets generated for this study are available on request to the corresponding author.

## Author Contributions

All the authors substantially have equally contributed to the development and preparation of the manuscript. Furthermore, all authors have approved the final version of the manuscript. Finally, the authors have agreed to be accountable for all aspects of the manuscript in ensuring that questions related to the accuracy or integrity of any part of it are appropriately investigated and resolved.

## Conflict of Interest Statement

The authors declare that the research was conducted in the absence of any commercial or financial relationships that could be construed as a potential conflict of interest.

## Footnotes

1 Item parceling consists in a procedure combining the items of a questionnaire into a smaller set of items to reduce the dimensionality and the number of parameters being estimated in the model, thus resulting in a more parsimonious measurement model and a more stable parameter estimate (Little et al., 2002).

2 The only exception was the factor loading of one of the measurement indicators of the latent variable “excitement seeking” which resulted significantly different across adult and older drivers (*t* = -2.56; *p* = 0.01).

## References

[B1] Af Wåhlberg A. E. Barraclough P. Freeman J. (2015). The driver behaviour questionnaire as accident predictor; a methodological re-meta-analysis. *J. Saf. Res.* 55 185–212. 10.1016/j.jsr.2015 26683562

[B2] Albarracin D. Shavitt S. (2018). Attitudes and attitude change. *Annu. Rev. Psychol.* 69 299–327. 10.1146/annurev-psych-122216-011911 28841390 PMC12479097

[B3] Anstey K. J. Wood J. Lord S. Walker J. G. (2005). Cognitive, sensory and physical factors enabling driving safety in older adults. *Clin. Psychol. Rev.* 25 45–65. 10.1016/j.cpr.2004.07.008 15596080

[B4] Barnard M. P. Chapman P. (2018). The moderating effect of trait anxiety on anxiety-related thoughts and actions whilst driving. *Pers. Individ. Differ.* 135 207–211. 10.1016/j.paid.2018.07.027

[B5] Beanland V. Sellbom M. Johnson A. K. (2014). Personality domains and traits that predict self-reported aberrant driving behaviours in a southeastern US university sample. *Accid. Anal. Prev.* 72 184–192. 10.1016/j.aap.2014.06.023 25075715

[B6] Broughton J. Brandstaetter C. Yannis G. Evgenikos P. Papantoniou P. Candappa N. (2012). *Basic Fact Sheet “The Elderly” Deliverable D3.9 of the EC FP7 Project DaCoTA*. Available at: http://www.dacota-project.eu/Deliverables/DaCoTA_WP3_D3_9%20rev.pdf

[B7] Caprara G. V. Barbaranelli C. Hahn R. Comrey A. L. (2001). Factor analyses of the NEO-PI-R inventory and the comrey personality scales in Italy and the United States. *Pers. Individ. Differ.* 30 217–228. 10.1016/S0191-8869(00)00030-1

[B8] Chen C.-F. (2009). Personality, safety attitudes and risky driving behaviors—Evidence from young Taiwanese motorcyclists. *Accid. Anal. Prev.* 41 963–968. 10.1016/j.aap.2009.05.013 19664433

[B9] Costa P. T. McCrae R. R. (1992). *Revised NEO Personality Inventory (NEO-PI-R) and NEO Five Factor Inventory (NEO-FF-I). Professional Manual.* Odessa: Psychological Assessment Resources Inc 10.1080/00140139.2015.1030460

[B10] de Winter J. C. Dodou D. Stanton N. A. (2015). A quarter of a century of the DBQ: some supplementary notes on its validity with regard to accidents. *Ergonomics* 58 1745–1769. 10.1080/00140139.2015.1030460 25777252

[B11] de Winter J. C. F. Dodou D. (2010). The Driver Behaviour Questionnaire as a predictor of accidents: a meta-analysis. *J. Saf. Res.* 41 463–470. 10.1016/j.jsr.2010.10.007 21134510

[B12] Deffenbacher J. L. (2016). A review of interventions for the reduction of driving anger. *Transp. Res. Part. F Traffic Psychol. Behav.* 42 411–421. 10.1016/j.trf.2015.10.024

[B13] Fishbein M. (2009). “An integrative model for behavioral prediction and its applica- tion to health promotion,” in *Emerging Theories in Health Promotion Practice and Research* eds Di Clemente R. J. Crosby R. A. Kegler M. C. (New York, NY: John Wiley & Sons) 215–234. 10.1111/j.1460-2466.2006.00280.x

[B14] Fishbein M. Cappella J. N. (2006). The role of theory in developing effective health communications. *J. Commun.* 56 S1–S17. 10.1111/j.1460-2466.2006.00280.x

[B15] Gidron Y. Gaygisiz E. Lajunen T. (2014). Hostility, driving anger, and dangerous driving: the emerging role of hemispheric preference. *Accid. Anal. Prev.* 73 236–241. 10.1016/j.aap.2014.09.011 25255416

[B16] Goldenbeld C. Levelt P. B. M. Heidstra J. (2000). Psychological perspectives on changing driver attitude and behaviour. *Recherche Transports Securite* 67 65–81. 10.1016/S0761-8980(00)90108-0

[B17] Ibm Corp. Released (2017). *IBM SPSS Statistics for Windows, Version 25.0.* Armonk, NY: IBM Corp 10.1016/S0191-8869(02)00010-7

[B18] Iversen H. Rundmo T. (2002). Personality, risky driving and accident involvement among Norwegian drivers. *Pers. Individ. Differ.* 33 1251–1263. 10.1016/S0191-8869(02)00010-7

[B19] Iversen H. Rundmo T. (2004). Attitudes towards traffic safety, driving behavior and accident involvement among the Norwegian public. *Ergonomics* 47 555–572. 10.1080/00140130410001658709 15204303

[B20] Källmen H. Wennberg P. Bergman H. (2011). Psychometric properties and norm data of the Swedish version of the NEO-PI-R. *Nord. J. Psychiat.* 65 311–314. 10.3109/08039488.2010.545433 21174492

[B21] Kim S. Hagtevt K. A. (2003). The impact of misspecified item parceling on representing latent variables in covariance structure modelling: a simulation study. *Struct. Equ. Model.* 10 101–127. 10.1207/S15328007SEM1001_5

[B22] Kock N. (2014). Advanced mediating effects tests, multi-group analyses, and measurement model assessments in PLS-based SEM. *Int. J. Collab.* 10 1–13. 10.4018/ijec.2014010101

[B23] Kock N. (2017). *WarpPLS User Manual Version 6.0. 2017.* Available at: http://cits.tamiu.edu/WarpPLS/UserManual_v_6_0.pdf

[B24] Kohn M. Schooler C. (1983). *Work and Personality: An Inquiry into the Impact of Social Stratification.* New York, NY: Norwood Ablex 10.1111/j.1559-1816.1997.tb01805.x

[B25] Lawton R. Parker D. Manstead A. S. R. Stradling S. G. (1997). The role of affect in predicting social behaviors: the case of road traffic violations. *J. Appl. Soc. Psychol.* 27 1258–1276. 10.1111/j.1559-1816.1997.tb01805.x

[B26] Little T. D. Cunningham W. A. Shahar G. Widaman K. F. (2002). To parcel or not to parcel: exploring the question, weighing the merits. *Struct. Equ. Model.* 9 151–173. 10.1207/S15328007SEM0902_1

[B27] Lucidi F. Giannini A. M. Sgalla R. Mallia L. Devoto A. Reichmann S. (2010). Young novice driver subtypes: relationship to driving violations, errors and lapses. *Accid. Anal. Prev.* 42 1689–1696. 10.1016/j.aap.2010.04.008 20728618

[B28] Lucidi F. Mallia L. Giannini A. M. Sgalla R. Lazuras L. Chirico A. (2019). Riding the adolescence: personality subtypes in young moped riders and their association with risky driving attitudes and behaviors. *Front. Psychol.* 10:300 10.3389/fpsyg.2019.00300 PMC6387963 30833922

[B29] Lucidi F. Mallia L. Lazuras L. Violani C. (2014). Personality and attitudes as predictors of risky driving among older drivers. *Accid. Anal. Prev.* 72 318–324. 10.1016/j.aap.2014.07.022 25108900

[B30] Magar E. C. Phillips L. H. Hosie J. A. (2008). Self-regulation and risk-taking. *Pers. Individ. Differ.* 45 153–159. 10.1016/j.paid.2008.03.014

[B31] Mallia L. Lazuras L. Violani C. Lucidi F. (2015). Crash risk and aberrant driving behaviors among bus drivers: the role of personality and attitudes towards traffic safety. *Accid. Anal. Prev.* 79 145–151. 10.1016/j.aap.2015.03.034 25823904

[B32] Marengo D. Settanni M. Vidotto G. (2012). Drivers’ subtypes in a sample of Italian adolescents: relationship between personality measures and driving behaviors. *Trans. Res. Part. F Traffic Psychol. Behav.* 15 480–490. 10.1016/J.TRF.2012.04.001

[B33] Martinussen L. M. Møller M. Grato C. G. (2014). Assessing the relationship between the driver behavior questionnaire and the driver skill inventory: revealing sub-groups of drivers. *Trans. Res. Part. F Traffic Psychol. Behav.* 26 82–91. 10.1016/j.trf.2014.06.008

[B34] Martinussen L. M. Møller M. Prato C. G. Haustein S. (2017). How indicative is a self- reported driving behaviour profile of police-registered traffic law offences? *Accid. Anal. Prev.* 99 1–5. 10.1016/j.aap.2016.10.031 27842281

[B35] Nordfjærn T. Jørgensen S. H. Rundmo T. (2010). An investigation of driver attitudes and behaviour in rural and urban areas in Norway. *Saf. Sci.* 48 348–356. 10.1016/j.ssci.2009.12.001

[B36] Nordfjærn T. ŞimŞekoglu Ö Can S. Somer O. (2015). Social cognition and personality traits related to risky driving in a Turkish sample. *J. Risk Res.* 18 452–466. 10.1080/13669877.2014.907330

[B37] Parker D. McDonald L. Rabbitt P. Sutcliffe P. (2000). Elderly drivers and their accidents: the aging driver questionnaire. *Accid. Anal. Prev.* 32 751–759. 10.1016/S0001-4575(99)00125-6 10994602

[B38] Parker D. Reason J. T. Manstead A. S. R. Stradling S. G. (1995a). Driving errors, driving violations and accident involvement. *Ergonomics* 38 1036–1048. 10.1080/00140139508925170 29105607

[B39] Parker D. West R. Stradling S. Manstead A. S. R. (1995b). Behavioural characteristics and involvement in different types of traffic accident. *Accid. Anal. Prev.* 27 571–581. 10.1016/0001-4575(95)00005-K 7546068

[B40] Pourabdian S. Azmoon H. (2013). The relationship between trait anxiety and driving behavior with regard to self-reported Iranian accident involving drivers. *Int. J. Prev. Med.* 4 1115–1121. 10.1080/00140139008925335 24319550 PMC3843297

[B41] Reason J. Manstead A. Stradling S. Baxter J. Campbell K. (1990). Errors and violations on the roads: a real distinction? *Ergonomics* 33 1315–1332. 10.1080/00140139008925335 20073122

[B42] Rothengatter T. Manstead A. S. R. (1997). “The role of subjective norm in predicting the intention to commit traffic violations,” in *Traffic & Transport Psychology, Theory and Application* eds Rothengatter T. Vaya Carbonell E. (Pergamon: Elsevier) 389–394. 10.1016/j.jsr.2007.04.005

[B43] Schwebel D. C. Ball K. K. Severson J. Barton B. K. Rizzo M. Viamonte S. M. (2007). Individual difference factors in risky driving among older adults. *J. Saf. Res.* 38 501–509. 10.1016/j.jsr.2007.04.005 PMC2186376 18023635

[B44] Šeibokaitë L. Endriulaitienë A. Sullman M. J. M. Markšaitytë R. Žardeckaitë-Matulaitienë K. (2017). Difficulties in emotion regulation and risky driving among Lithuanian drivers. *Traffic Inj. Prev.* 18 688–693. 10.1080/15389588.2017.1315109 28384034

[B45] Shen B. Qu W. Ge Y. Sun X. Zhang K. (2018). The relationship between personalities and self-report positive driving behavior in a Chinese sample. *PLoS One* 13:e0190746 10.1371/journal.pone.0190746 PMC5764283 29324823

[B46] Steinberg L. Albert D. Cauffman E. Banich M. Graham S. Woolard J. (2008). Age differences in sensation seeking and impulsivity as indexed by behavior and self-report: evidence for a dual systems model. *Dev. Psychol.* 44 1764–1778. 10.1037/a0012955 18999337

[B47] Taylor J. E. Deane F. P. Podd J. V. (2007). Driving fear and driving skills: comparison between fearful and control samples using standardised on-road assessment. *Behav. Res. Ther.* 45 805–818. 10.1016/J.BRAT.2006.07.007 16962560

[B48] Tenenhaus M. Vinzi V. E. Chatelin Y. M. Lauro C. (2005). PLS path modeling. *Comput. Stat. Data Anal.* 2005 159–205. 10.1016/j.csda.2004.03.005

[B49] Ulleberg P. Rundmo T. (2003). Personality, attitudes and risk perception as predictors of risky driving behaviour among young drivers. *Saf. Sci.* 41 427–443. 10.1016/S0925-7535(01)00077-7

[B50] Wijnen W. Stipdonk H. (2016). Social costs of road crashes: an international analysis. *Accid. Anal. Prev.* 94 97–106. 10.1016/j.aap.2016.05.005 27269998

[B51] Wong I. Y. Mahar D. Titchener K. (2015). Driven by distraction: investigating the e?ects of anxiety on driving performance using the attentional control theory. *J. Risk Res.* 18 1293–1306. 10.1080/13669877.2014.919516

[B52] World Health Organization [WHO] (2018). *Road Traffic Injury: Key Facts.* Available at: http://www.who.int/news-room/fact-sheets/detail/road-traffic-injuries

[B53] Yang J. Du F. Qu W. Gong Z. Sun X. (2013). Effects of personality on risky driving behavior and accident involvement for chinese drivers. *Traffic Inj. Prev.* 14 565–571. 10.1080/15389588.2012.748903 23859184

[B54] Zhang T. Chan A. H. S. Zhang W. (2015). Dimensions of driving anger and their relationships with aberrant driving. *Accid. Anal. Prev.* 81 124–133. 10.1016/j.aap.2015.05.005 25984643

[B55] Zicat E. Bennett J. M. Chekaluk E. Batchelor J. (2018). Cognitive function and young drivers: the relationship between driving, attitudes, personality and cognition. *Trans. Res. Part. F Traffic Psychol. Behav.* 55 341–352. 10.1016/j.trf.2018.03.013

